# Assessment of attitudes and targeted educational needs for refugee care providers in a Ugandan hospital

**DOI:** 10.5116/ijme.5b64.9630

**Published:** 2018-08-24

**Authors:** Achille Bapolisi, Katherine Crabtree, Jana Jarolimova, Caitrin Kelly, Katherine Kentoffio, Palka Patel, Geren Stone, Vincent Batwala

**Affiliations:** 1 Mbarara University of Science and Technology, Department of Psychiatry, Uganda; 2 Massachusetts General Hospital (MGH) Global Medicine, USA; 3 Mbarara University of Science and Technology, Directorate of Research and Graduate Training, Uganda

**Keywords:** Refugee health, medical education, sub-Saharan Africa, curriculum development, Uganda

## Abstract

**Objectives:**

To evaluate medical trainees’ attitudes toward refugee patients in a refugee host country, and to identify educational needs.

**Methods:**

A 54-question cross-sectional questionnaire was administered to a convenience sample of 81 post-graduate medical trainees at Mbarara Regional Referral Hospital, Uganda, in 2016. Descriptive statistics on medical trainees’ attitudes and educational needs regarding care for refugees were calculated. One-way ANOVA was used to assess relationships between an attitude scale and respondent characteristics. Reliability and validity of the survey items and attitude scale were assessed using Cronbach’s alpha, item-to-scale correlation, and factor analysis.

**Results:**

The mean score on the attitude scale of 2.8 (SD=1.7) indicated positive attitudes toward refugees. All respondents had contact with refugees, and 89% (n=72) reported a need for further training. Many specifically indicated the need for training in use of translators, support personnel, and behavioral health. Cronbach’s alpha values of greater than 0.7 indicated good internal consistency. Item-to-scale correlation and factor analysis validate the use of an attitude scale. ANOVA showed no significant difference between mean attitude scores in gender (F_(1,77)_=0.11, p=0.7367), country of origin (F_(1.78)_ =0.53, p=0.8723), or year of study (F_(4,74) =_0.31, p=0.8273).

**Conclusions:**

Medical trainees in Uganda report positive attitudes toward refugees and a need for additional education in refugee care in multiple specific areas. This study piloted the use of an attitude scale for refugee healthcare providers with promising validity and reliability. Use of these questions could inform curriculum development in refugee host countries.

## Introduction

In the ongoing global refugee crisis, sub-Saharan African (SSA) nations are on the front lines as host countries for those fleeing violence.^1^ Any hospital physician in SSA may care for refugee patients, as the role of host countries includes provision of healthcare to refugees both within refugee settlements and in hospitals outside of settlements.^2^^-^^5^ A refugee population presents a unique set of challenges to healthcare providers including health disparities, uncommon diagnoses, language barriers, and different patterns of hospital utilization.^6^^-^^7^ These challenges underscore the need to incorporate refugee health education into medical curriculum to ensure that refugees receive optimum care.

Educational needs of medical providers caring for refugees have primarily been studied in resettlement countries in Europe, North America and Australia.^8^^-^^15^ Dias and colleagues found that in Portugal, physicians’ attitudes toward immigrant patients worsened as they spent more time with those patients, while their knowledge level regarding particular barriers to care for this population remained low.^8^ This study and many others have concluded that ongoing education is needed for refugee care providers in resettlement countries. ^9^^-^^15^

Most research on refugee health comes from resettlement countries, but it is difficult to extrapolate these findings to refugee host countries. Host countries differ from refugee resettlement countries in important respects. In host countries, refugees live in settlements or camps rather than being permanent residents or citizens of the country. Many host countries have significantly different healthcare infrastructure and medical education from resettlement countries.^16^^-^^17^ In SSA, both attitudes and educational needs may be different from those in the resettlement countries. To our knowledge, only one study in the field of trauma-informed care by McDonald and colleagues has reported on provider perspectives in host countries.^18^

We completed a study with the aim to develop and validate a questionnaire to assess attitudes of Mbarara’s postgraduate medical trainees toward refugees and measure their specific educational needs.

## Methods

### Study design and Participants

We conducted a cross-sectional, questionnaire-based pilot study to assess the need for curriculum development around issues of refugee healthcare. The target population consisted of post-graduate medical trainees caring for refugees in a refugee host country. Post-graduate trainees in this paper are defined as those who have finished their medical school curriculum and continue to participate in regular didactics while providing supervised care to patients. Permission was obtained from instructors, and department heads and participation were voluntary.   No identifying information was collected. The study was approved by the Mbarara University of Science and Technology Research Ethics Committee, the Massachusetts General Hospital Institutional Review Board, and the Ugandan National Council for Science and Technology.

### Setting Mbarara

The study was completed at Mbarara University of Science and Technology in Mbarara, Uganda.  Participants were from clinical training departments including anesthesia, dermatology, internal medicine, obstetrics/gynecology, ophthalmology, otolaryngology, pediatrics, psychiatry, and surgery. The regional hospital serves as a referral facility to a large refugee population from Nakivale settlement, which is populated by more than 100,000 refugees.^19^ The current curriculum for physicians in training at this facility does not include didactics related to refugee health care.

### Data collection

The questionnaire was administered to all postgraduate trainees present during required didactic sessions at the departments listed above. Paper copies of questionnaires and consent forms were provided to participants and were self-administered in English. The research team collected completed questionnaires and consent forms within 24 hours.

### Procedures

Initial development of the questionnaire included literature review for general survey development, themes of the survey and eight previously used questions for measurement of attitudes toward refugees.^7^^-^^16^^,^^20^^,^^21^ Two content-area experts assessed the questionnaire for content validity, relevance, and comprehensiveness. The questions were evaluated by Ugandan English speakers to assess for clarity and any need for back-translation.  The questionnaires were administered to one former and one current trainee for comprehension, clarity and readability prior to administration.  The final questionnaire consisted of 54 questions, 42 on a Likert scale and the remainder open-ended. Six questions covered demographics. Thirty-three questions on a Likert scale covered knowledge of logistical details of inpatient refugee care including use of translators, United Nations High Commissioner for Refugees (UNHCR) ancillary staff, psychiatric services, attitudes toward refugees, barriers to care for refugees, and need for further education.

### Data analysis

Evaluation of the questionnaire included an examination of internal consistency using Cronbach’s alpha; item to scale correlation measurement for questions assessing attitudes of providers; and use of factor analysis. Item to scale correlation is a tool to measure the strength of association of individual items to an overall scale.  We used factor analysis to further evaluate the validity of the attitude assessment scale.  Factor loadings of greater than 0.4 were considered meaningful.   We confirmed the applicability of factor analysis using Kaiser-Meyer-Okin and Bartlett tests. Multiple methods were used to confirm the retention of one latent factor, including the use of Eigenvalues greater than 1 and scree plot. Oblique rotation was used to rotate the factors given the high likelihood of correlation between the variables, and rotated loading plot was used to verify visually. This latent factor was labeled the “attitude scale”. A component score for the scale was made using equally weighted variables. Positive responses on the Likert scale yielded one point per question, and negative or neutral responses on the Likert scale yielded zero points.

We measured descriptive statistics including percentages, means, standard deviations and ranges where appropriate. One-way analysis of variance was used to assess the relationship between the attitude scale scores and independent variables including gender, year of practice and country of origin.   Data were analyzed using STATA version 14.

## Results

Post-graduate trainees who participated had a range of <1-8 years of experience in medical practice. The average age of participants was 30.1 years with a range of 22-44; 72% (n=58) were male, and 72% (n=58) were Ugandan.  Other countries of origin included Burundi, Democratic Republic of Congo, Kenya, Rwanda, Sierra Leone, Somalia and South Sudan. The full demographic data of the participants is provided in Table 1. Most respondents (98%, n=79) reported having provided care to a refugee patient, and on average reported caring for 30 (SD=25.7) refugee patients monthly, with a range of 2-100 per month.

**Table 1:** Demographics of respondents, Mbarara, Uganda, December 2016 (N=81)

Variable		
Age (Mean, SD)		30.1, 4.8
Range (Years)		22-44
		% (n)
Gender		
	Female	28 (23)
	Male	72 (58)
Nationality		
	Ugandan	72 (58)
	Other^*^	28 (23)
Field		
	Intern (undifferentiated)	17.7 (15)
	Internal Medicine	11.4 (9)
	Obstetrics/Gynecology	25.3 (21)
	Pediatrics	7.6 (6)
	Psychiatry	6.3 (5)
	Surgery	19.0 (15)
	Other	12.7 (10)

^*^Other countries of origin include Burundi, Democratic Republic of Congo, Kenya, Rwanda, Sierra Leone, Somalia, and South Sudan

The reliability of the 41 items on the Likert scale was assessed using Cronbach’s alpha and was 0.72, which indicates good reliability.

Item-to-scale correlation and subsequently factor analysis suggested the presence of a latent factor in the “attitude questions” obtained from the literature review for assessment of attitudes related to refugee care, with retention of seven of the eight items used in the Portuguese study.^8^ The item-to-scale correlation showed that all of these items had adjusted correlations above the acceptable lower bound of 0.3. The Cronbach’s alpha value for the latent attitude factor was 0.7859, again indicating good reliability. The KMO and Bartlett’s test for correlation of the variables included in the summary scores shows KMO >0.6 for all subjects and a significant Bartlett’s test of sphericity, indicating that it is acceptable to assess the data using factor analysis. The attitude factor accounts for 42% of the variance (Table 2).

**Table 2:** Refugee health education needs assessment responses, Mbarara, Uganda, December 2016 (N=81)

Specific points of interest	Affirmative responses % (n)
Know how to contact UNHCR liaison	59 (48)
Have contacted the UNHCR liaison in the past	67 (54)
Ask refugees about trauma history	23 (19)
Use translators when needed	40 (32)

The mean score on the resulting attitude scale was 2.8 (SD=1.9). One-way ANOVA was used to compare the resulting attitude scale scores across year of study, gender and country of origin.  This showed no significant difference between mean attitude scores across gender (F_(__1,77)_=0.11, p=0.7367), country of origin (F_(1.78)_=0.53, p=0.8723), or year of study (F_(4,74)_=0.31, p=0.8273).

In response to specific questions regarding prior medical education on refugee care, only 13% (n=10) of respondents reported having received information from school or courses. Most respondents 67% (n=55) reported obtaining knowledge about refugees from media, patients, and colleagues; and 59% (n=48) reported previous personal experience with refugees as an information source. The majority of respondents 86% (n=70) were in favor of further medical education on refugee care. Only 13.8% (n=11) reported that they would not benefit from further education.

In response to specific questions regarding future medical education on refugee care, interesting points included that 41% (n=33) indicated that they do not know how to contact the UNHCR nurse liaison and 33% (n=27) reported that they had never communicated with the nurse liaison regarding their refugee patients. In addition, 74%(n=60) of respondents reported that it is more difficult to communicate with refugee patients than Ugandan patients, but 60% (n=49) of respondents reported that they do not always use translators provided by UNHCR.

Of all respondents, 40% (n=32) agreed that their differential diagnosis changes when a patient has a history of trauma, and 52% (n=43) of respondents believed that refugee patients have experienced more recent trauma than most Ugandan patients. However, only 23% (n=19) reported asking patients about their history of psychological trauma. Among the respondents, 40% (n=32) reported that they obtain psychiatry consult in a patient with a history of psychological trauma, with only 51% (n=41) reporting that it is easy to obtain a psychiatry consults. Highlights of these results are reported in Table 3.

**Table 3:** Item-Scale Correlation and Factor Analysis with communalities of each item

Item	Adjusted Item-Scale Correlation	Loading	h^2^	Mean	SD
Refugees use more resources than Ugandans.	0.45	0.6	0.54	2.9	0.12
Some refugees are dangerous and aggressive.	0.63	0.71	0.61	2.7	0.12
Some refugees take advantage of the social benefits Uganda offers them	0.49	0.57	0.45	3.1	0.12
Some refugees dramatise and exaggerate their problems to facilitate the process of resettlement	0.50	0.58	0.53	3.3	0.12
Frequently refugees do not respect the health services' rules.	0.58	0.7	0.61	2.6	0.1
When refugees are admitted to my hospital service they behave like victims.	0.45	0.58	0.53	2.8	0.1
Refugee patients are generally attentive to hospital counseling and instructions	0.45	0.51	0.66	3.3	0.1
Variance%		42			
KMO		0.73			
Bartlett's		0			

## Discussion

This pilot study represents a first attempt at describing the attitudes and educational needs of medical trainees toward refugees in a host country. Other studies on the topic have been completed exclusively in high-income refugee resettlement countries.

The study demonstrates key differences between the setting of the host country and the resettlement country. Importantly, it documents the universality of care for refugees among the trainees at this institution, whereas studies in resettlement countries note that a majority of providers do not see refugees.^7^ Additionally, resettlement country studies have largely reported on health care provider populations which are culturally distinct from the refugee population.^18^   In contrast, this study demonstrates that 28% of the medical trainees were themselves from the same countries as refugees, including Burundi, Democratic Republic of Congo,  Somalia, and South Sudan. Educational needs around cultural competency may, therefore, be different in this setting.

Multiple studies have indicated that identifying attitudes toward the refugee population is important in gauging the reception of and need for further training.^9^^-^^13^ Previously, attitudes among medical trainees in SSA host countries toward refugees had not been assessed. This study is the first to show that medical trainees in an SSA institution generally have positive attitudes toward refugees. The study is the first to attempt to validate attitude questions to create an “attitude scale” in a host country, and the validity and reliability of the results support the use of these questions in this setting, although testing in a larger population is needed.

In terms of general educational need, we found that participants were receptive to education on refugee health. Most trainees have some personal experience with refugees but report little formal education on healthcare for refugees during their training.  The majority of respondents were also “learning on the job,” as they reported patients and previous personal experiences as frequent sources of information on refugee care. This is consistent with findings of studies in resettlement countries.^8^

The study documented that, similar to their colleagues in resettlement countries, medical trainees in this institution need additional training and encouragement in use of translators. The importance of translators in communication with patients has been well-documented in resettlement settings, and training in use of translators is important to maximize their efficacy.^22^^,^^23^

A key difference in training needs between host and resettlement countries is that in host countries, interaction of healthcare providers with UNHCR resources is crucial. UNHCR facilitates care of refugees at many hospitals using a nurse liaison. Contact with the nurse liaison is critical for the refugee to obtain funding for all medication and diagnostic tests as well as a stipend for food while in the hospital. Our study demonstrates that additional education is needed in this area as not all of the medical providers had interacted with the nurse liaison. This need should be studied across programs where providers must interact with UNHCR to provide quality care, as it could represent potential for tangible quality improvement in the care of refugees in host countries. The coordination of care with psychiatry or behavioral health is important, as many refugees continue to suffer the sequelae of violence from which they have fled. This study documented low comfort levels with addressing histories of trauma, consulting psychiatry, and making referrals to psychiatric services. While this is also an important topic in resettlement countries, this is a topic that has been studied in the patient population in Uganda and noted to be highly prevalent.^24^

Our study has several limitations. First, the study was done in one teaching hospital, thus limiting the generalizability of these results to a larger region. Potential recall bias limits the questionnaire. The small sample size limits subgroup analysis. The small sample size also impacts the use of factor analysis. However, the strength of item-to-scale correlation for the attitude scale supports the use of factor analysis.

In summary, we found that these providers are receptive to additional education on refugee health. Questions used to assess provider attitudes in the resettlement setting performed well in this setting and indicated that these providers have positive attitudes toward their refugee patients. Several areas of educational need among medical trainees in an SSA refugee host country mirror those reported in refugee resettlement countries, but there are also key differences.

Based on our results, we would recommend that a curriculum designed to enhance healthcare provision to refugees in host countries include education on the use of translators, elicitation of the trauma history and subsequent referral to psychiatric services, and education on logistics of interacting with UNHCR resources. While our results are not necessarily generalizable to the remainder of SSA host countries, we would recommend assessment and consideration of these factors at other training institutions that are considering a curriculum on this important subject.

### Acknowledgments

Alice Alaso, UNHCR, MTI Nakivale; Dean Sam Maling MD, MUST; Bourse d’Etude du Professeur Bringmann aux Universités Congolaises (BEBUC); German Academic Exchange Service (DAAD); Amir Abdallah MD, MUST; Charles Abonga MD, MUST; Kelli O’Laughlin, MD, MGH; Mark Siedner MD, MGH; Hang Lee PhD, MGH; Karen Donelan ScD, MGH; Mark Blais MD PhD, MGH.

### Conflict of Interest

The authors declare that they have no conflict of interest.
